# Deep‐learning power and perspectives for genomic selection

**DOI:** 10.1002/tpg2.20122

**Published:** 2021-07-26

**Authors:** Osval Antonio Montesinos‐López, Abelardo Montesinos‐López, Carlos Moises Hernandez‐Suarez, José Alberto Barrón‐López, José Crossa

**Affiliations:** ^1^ Facultad de Telemática Universidad de Colima Colima Colima 28040 México; ^2^ Departamento de Matemáticas, Centro Universitario de Ciencias Exactas e Ingenierías (CUCEI) Universidad de Guadalajara Guadalajara Jalisco 44430 México; ^3^ Facultad de Ciencias Universidad de Colima Colima Colima 28040 México; ^4^ Department of Animal Production (DPA) Universidad Nacional Agraria La Molina Av. La Molina s/n La Molina Lima 15024 Perú; ^5^ Colegio de Postgraduados Montecillos, Edo de México 56230 México; ^6^ Biometrics and Statistics Unit, Genetic Resources Program International Maize and Wheat Improvement Center (CIMMYT) Km 45, Carretera Mexico‐Veracruz, Edo. De Mexico DF 52640 Mexico

## Abstract

Deep learning (DL) is revolutionizing the development of artificial intelligence systems. For example, before 2015, humans were better than artificial machines at classifying images and solving many problems of computer vision (related to object localization and detection using images), but nowadays, artificial machines have surpassed the ability of humans in this specific task. This is just one example of how the application of these models has surpassed human abilities and the performance of other machine‐learning algorithms. For this reason, DL models have been adopted for genomic selection (GS). In this article we provide insight about the power of DL in solving complex prediction tasks and how combining GS and DL models can accelerate the revolution provoked by GS methodology in plant breeding. Furthermore, we will mention some trends of DL methods, emphasizing some areas of opportunity to really exploit the DL methodology in GS; however, we are aware that considerable research is required to be able not only to use the existing DL in conjunction with GS, but to adapt and develop DL methods that take the peculiarities of breeding inputs and GS into consideration.

## Core Ideas

- Use of deep learning (DL) in genomic selection (GS)
- To capture patterns in the data by a nonlinear transformation
- How to adapt DL to plant breeding and GS?
- Explore DL power and perspectives for genomic selection

**Abbreviation**
DL: deep learning
GAN: generative adversarial network
GS: genomic selection
VAE: variational auto‐encoder.

## 1 INTRODUCTION

The use of prediction models is a key component for the successful implementation of genomic selection (GS), which is considered a predictive methodology used to train models with a reference population containing known phenotypic (output) and genotypic (input) data to perform predictions for a testing data set that only contains genomic (input) data. However, because a universal model is nonexistent (no‐free‐lunch theorem), it is necessary to evaluate many models for a particular data set and subsequently choose the best option for each particular situation. The no‐free‐lunch theorem more or less states that there is no perfect statistical machine‐learning method that will perform well at any problem. For every problem, a certain algorithm is suited and achieves good results, while other methods fail heavily (Wolpert & Macready, 1997, 2005). For this reason, a great variety of statistical (ridge regression, mixed‐models, Bayesian regression, generalized regression, etc.) and machine‐learning (support vector machine, random forest, etc.) models are used for prediction in GS.

In the context of plant science, the most popular statistical learning models are (a) the linear mixed model, which uses Henderson's equations (Henderson, 1950, 1975) to find the best linear unbiased estimates for fixed effects, as well as the best linear unbiased predictors for random effects and (b) the Bayesian counterpart of this model, which has different versions [Bayesian Ridge regression, BayesA, BayesB, BayesC, Bayes Lasso, etc.] (Gianola, 2013; Kärkkäinen & Sillanpää, 2012; Meuwissen et al., 2001). The most popular methods of machine learning are random forest and support vector machines, which are very easy to implement since they require few hyperparameters to be tuned and little time to provide very competitive predictions. From here onward, we will refer to those methods arising from statistical and machine‐learning fields as statistical machine‐learning methods.

Most of the above‐mentioned statistical machine‐learning methods require highly preprocessed inputs (feature engineering) in order to produce reasonable predictions. Feature engineering is the action of using discipline knowledge to extract features from raw data. These features can be used to increase the performance of statistical machine‐learning algorithms. Feature engineering can be treated as applied machine learning itself (Chollet & Allaire, 2017). In other words, most statistical machine‐learning methods need more user intervention to preprocess inputs, which must be done manually. However, those models under the umbrella of deep learning (DL) are more robust; they can perform automatic feature engineering and are more powerful at capturing more complex patterns in the input data since they are a generalization of artificial neural networks where more than one hidden layer is included in the model.

**Core Ideas**

- Use of deep learning (DL) in genomic selection (GS)
- To capture patterns in the data by a nonlinear transformation
- How to adapt DL to plant breeding and GS?
- Explore DL power and perspectives for genomic selection

An artificial neural network is a system composed of many simple elements of processing that operate in parallel and whose function is determined by the structure of the deep network and the weight of connections, where the processing is done in each of the nodes or computing elements that has a low processing capacity (Francisco‐Caicedo & López‐Sotelo, 2009). For this reason, DL models perform so‐called representation learning (also called feature learning). This means that a model learns new and improved representations with regard to the raw data because the learning process is done in multiple steps through multilevel transformations (applying many hidden layers) (LeCun et al., 2015). Additionally, its power is attributed, in part, to the fact that nonlinear transformations are performed between subsequent layers (Duda et al., 2000) and the learning process is done via training data (LeCun et al., 2015) like all machine‐learning methods. Because of this, DL models are very flexible and promise to extract knowledge in data‐driven fashion from large datasets while requiring limited domain expertise (Eraslan et al., 2019).

However, applications of DL models are still not generalized, as exemplified by Emmert‐Streib et al. (2020a), who pointed out that most of these applications are in computer science (52.1%) and engineering (41.5%) and less in the fields of medical imaging (6.2%), robotics (2.6%), and computational biology (2.5%). In biology, DL applications are gaining increasing momentum in predicting the structure and function of genomic elements such as enhancers, promoters, chromatin interaction prediction (Singh et al., 2019; Whalen et al., 2016; Zeng et al., 2018), and gene expression levels. However, DL continues to be infrequently used in GS, in part because its superiority in predicting performance over conventional statistical machine‐learning methods (Montesinos‐López et al., 2021) is unclear. In addition, DL methods require larger data sets and considerably more computational resources for their successful implementation.

The limited number of applications of DL for GS shows that there is huge potential for these models to improve the selection of candidate genotypes at an early stage as well as improving the understanding of the complex biological process involved in the relationship between phenotypes and genotypes. In part, this potential can be attributed to the way these models are built, which gives them the power to capture more complex patterns in data. For this reason, in this paper we explore DL power and perspectives for GS in addition to the obstacles for its successful implementation in plant breeding programs.

## 2 DEEP LEARNING AS A SPECIAL STATISTICAL MACHINE‐LEARNING METHOD

As mentioned before, DL is a statistical machine‐learning method that is considered one of the best, if not the best, algorithms for dealing with perceptual problems such as image classification. This mostly is due to the fact that DL models work with multiple hidden layers (for more details, see Montesinos‐López et al., 2021) that increase their power to capture better nonlinear patterns in the data. For this reason, the ‘deep’ in DL models stands for the successive layers of representations (Chollet & Allaire, 2017). Deep learning models need to be understood not as a single method but as a family of learning algorithms that is nowadays very popular for prediction and association tasks that use multilayer neural networks with many hidden units in common (LeCun et al., 2015). The first big moment of DL methods was in 2012 for classifying high resolution color images into 1000 different categories using a training set of 1.4 million images. During this intervention, 83.6% of all images were correctly classified, which outperformed the benchmark (74.3%) with the same data set in 2011 using classic approaches (Chollet & Allaire, 2017).

However, in 2015, while using this same data set, 96.4% of all total images were correctly classified. For this reason, DL is now used for complex tasks like (a) automated driving, as it is able to detect objects such as pedestrians and stop signs; (b) playing AlphaGo with Robots trained with DL methods that outperform human champions; (c) using an algorithm to classify people with skin cancer, that performs similarly to dermatological experts (Brinker et al., 2019); (d) analyzing particle data as the European Organization for Nuclear research (CERN) did to replace a classic machine‐learning method (decision‐tree based) with DL methods (Chollet & Allaire, 2017); and (e) for predicting what shapes proteins fold into, also known as the ‘protein folding problem’ that has been a great challenge in biology for the past 50 yr. In this particular problem, the results from the 14th critical assessment of protein structure prediction show an overall accuracy across all targets, reaching 92.4 in the global distance test on a 0‐to‐100 scale (Kaplan & Haenlein, 2019). Although this list of tasks is not comprehensive, it shows that DL applications have the potential to changing many current paradigms.

### 2.1 Why use DL in GS?

The main reasons for using DL in GS included the following: (a) DL is more powerful in capturing complex patterns in the data because of the inclusion of many neurons communicated in complex ways and multiple nonlinear transformations through hidden layers; (b) DL supports raw (not preprocessed) inputs, which is impossible in most statistical machine‐learning methods; (c) DL supports varied inputs that can accommodate pedigree, genomic data, environmental data, and other omics data (metabolomics, microbiomics, phenomics, proteomics, transcriptomics, etc.); (d) DL is more efficient for large and complex data sets than most statistical machine‐learning methods (Montesinos‐López et al., 2021); and (e) DL is very flexible and its network architecture permits a “Lego‐like” construction of new models while an unlimited number of neural network models can be constructed by using elements of the core architectural building blocks of existing DL models (Montesinos‐López et al., 2021).

### 2.2 Why can research in DL be applied to GS?

As pointed out before, DL methods have been used for the development of successful technological products like autonomous vehicles as well as face and voice recognition systems, where DL outperforms most machine‐learning models. However, the training process for each particular technological product is very specific (tailored to suit) and large amounts of resources are spent on its development. Since thousands of inputs (images and other inputs) are collected, DL looks for, by trial and error, the best architecture (topology) and tuning parameters, thus making it valid only for one particular problem. This means that the tuning process is quite expensive and time consuming, as it requires huge computational resources.

Nevertheless, it is still unclear if DL methods outperform conventional statistical machine‐learning methods for GS (Montesinos‐López et al., 2021). This can be attributed to the following issues: (a) most of the applications so far have used small or moderate data sets; (b) the tuning process is quite limited since only small grids of hyperparameter combinations are evaluated because of the lack of computer resources; (c) the applications use conventional architectures (mostly fully connected networks and convolutional neural networks) and activation functions of deep neural networks; (d) most of the inputs are limited to markers and environmental information; (e) many times, the inputs must be preprocessed using linear models that are unable to retain the complex patterns; and (f) because of the lack of expertise in using these methods and the fact that most breeders see the models as a black box to which they provide inputs and obtain outputs, there is little time dedicated to model calibration. This last point is important because the training process in DL is most time consuming; you must experiment extensively as opposed to only evaluating the factors believed to improve the prediction performance.

As such, many other factors must be evaluated and some examples are changing the architecture design, depth, width, pathways, weight initialization, loss function, etc. This implies that many of the hyperparameters must be evaluated in order to learn the effect of their increase or decrease. It is also necessary to evaluate a large range of learning rates and other hyperparameters to learn the efficiency of the behavior of the network over a large range of learning rates and hyperparameters. Many times, even after doing all this, the result is insufficient, and data augmentation must be used in combination with regularization methods (dropout, Ridge and Lasso regularization) to increase the training set. In this sense, the generalization of the model can be improved.

Yet another reason why it is not possible to claim superiority of DL models over conventional statistical machine‐learning methods is that the transfer learning approach of DL has not been used in GS since it cannot be easily transferred to other domains (like GS) because of the very different type of inputs. This is because the information content of one data set does not have the same meaning for each data type and for each application domain (Emmert‐Streib et al., 2020a, 2020b). Finally, the prediction performance of statistical machine‐learning methods depends on the genetic architecture of the trait. Under purely additive actions, conventional statistical learning methods outperformed machine‐learning approaches. However, when there was nonadditive action, predictive ability depended on the number of loci controlling the trait (Abdollahi‐Arpanahi et al., 2020).

Nonetheless, DL methods offer a range of possibilities (areas of opportunity) that can be successfully used in GS. However, this requires additional research using a family of models for the following:

1. Modifying, adapting, or inventing new DL architectures, activation functions, and tuning strategies for the specific context of GS.
2. Adapting, improving, and developing more user‐friendly software for DL applications in GS, aiming to ease data acquisition and model evaluation in such a way that the user only provides the inputs to obtain satisfactory predictions. This is important since even though there has been a great deal of improvement in the existing software for DL, one must still possess considerable programing skills.
3. Performing greater benchmarking studies to compare the prediction performance of existing DL methods with those that are very popular in GS in such a way as to promote the use of those algorithms with good results while being able to improve those that are not quite as good. This is very important since we cannot blindly adopt DL methods because if they are not tuned correctly, they may produce wrong recommendations. Furthermore, many times DL is not the right method for the data set and should not be hastily employed only because it seems sophisticated.
4. For exploring transfer learning for GS. The goal of transfer learning is to use the knowledge learned from one specific set of environments to ease the learning tasks in another different but quite similar environment. That is, the key idea behind transfer learning is that a data set from a different field can be the starting point for training a predictive model. Usually the model trained with a large data set (for example, with natural images) is transferred to a target model, with a small data set, that will perform similar tasks but in a different field, for example, in medical imaging (Koumakis, 2020). This is very important since, unfortunately, the assessment of DL models needs to be conducted in a domain‐specific manner, as the transfer of knowledge between such models is not straightforward. The learnable parameters in the pretrained model are reused in the new model as the feature extractor. The learnable parameters in the new model will be trained on a significantly smaller data set. In this sense, transfer learning alleviates the demand for larger data sets while still producing an accurate model (Liu et al., 2020).

Transfer learning has been used successfully for image classification problems and saves considerable resources in labeling since the training data set is only moderate. However, to successfully use transfer learning, the two problems to be solved should be closely related. The idea is to share the available learnable parameters in the pretrained model with the new model that we want to train with a small data set. This method is very attractive because, if successful, then DL methods can be used in small data sets. The pretrained model must be trained with large data sets in order to solve the problem of deficient sample sizes. Additionally, both problems should be similar to be able to use the learned parameters in the pretrained model. For example, the learnable parameters used for maize (*Zea mays* L.) prediction with some species using a large data set can be used as a pretraining model for maize prediction with small data sets of other species of maize. Transfer learning applications in plant breeding are just starting. For example, Meng et al. (2021) used it for predicting transcriptional responses to cold stress across plant species; they found that models trained with data from one species successfully predicted which genes would respond to cold stress in other related species. Cross‐species predictions remained accurate when training was performed in cold‐sensitive species and predictions were performed in cold‐tolerant species and vice versa. Also, the applications of transfer learning for cancer survival prediction using gene expression data are just starting (López‐García et al., 2020).

5 For exploring how to use reinforcement learning in the context of GS. Reinforcement learning is a subfield of machine learning that teaches an agent how to choose an action from their action space, within a particular environment, in order to maximize rewards over time. For instance, when playing the Atari game, the computer or agent playing this game was positively rewarded when the outcome of the game was positive based on the actions performed. The algorithm was able to learn some of the games to a level where it performed better than humans (Patterson & Gibson, 2017). Reinforcement learning has four essential elements: (a) agent—the program you train with the goal of doing the work you specify; b) environment—the world, real or virtual, in which the agent performs actions; (c) action—a movement made by the agent that causes a change of state in the environment; and (d) reward—the evaluation of an action, which can be positive or negative. Under this method, the training system gives the agent input from the environment and rewards the agent when the outcome is positive. Many times, actions will affect not only the immediate reward but also future rewards. The mechanics of trial and error and delayed rewards are key features of reinforcement learning. Nowadays, reinforcement learning has found application in problems from robotics to games and healthcare (Arulkumaran et al., 2017; Mnih et al., 2015).
6 For exploring deep generative models (generative adversarial networks [GANs] and variational auto‐encoder [VAE] methods) to generate new inputs (fictitious markers or independent variables) that are indistinguishable from the original training set. These methods are very efficient at creating fake images (or text) that, for humans, are identical to the real ones. In biology, these methods are being used to generate artificial genomes; fake DNA, such as microbial genomes (Nielsen & Voigt, 2018 ); sequences (Linder et al., 2019; Liu et al., 2020; Yelmen et al., 2021 ); single‐cell RNA sequencing data (Grønbech et al., 2020; Liu et al., 2020; Marouf et al., 2020 ); protein sequences (Repecka et al., 2021; Sinai et al., 2017 ); promoter sequences (Y, Wang et al., 2020); high‐resolution Hi‐C data (Hong et al., 2020; Liu et al., 2019b; Liu et al., 2020 ); among others. Also, VAE in GS has been applied to visualize population structure (Battey et al., 2021). Deep generative models (GAN and VAE) belong to unsupervised methods that efficiently learn complex data distributions. The use of these models is very promising for crop improvement as a way of creating new DNA elements, artificial genomes, or even regulatory circuits with desirable functions (H. Wang et al., 2020b). However, as a reviewer pointed out, there is still a long way to go to be able to create a synthetic genome for complex plants since some applications done until now are for a single‐celled organism.
7 For training or retraining breeders and people involved in genomic prediction in these new frameworks for DL, as exemplified by Keras (Chollet & Allaire, 2017). These new frameworks allow you to train with conventional and large data sets, univariate and multivariate linear regression models, generalized regression models (with families: Poisson, multinomial, Binomial and Gaussian), conventional artificial neural networks (with one hidden layer); and state‐of‐the art DL models with as many hidden layers as you want.
8 For exploring the deep compression methods in GS to reduce the computation and storage required by neural networks. These methods are very promising since nowadays DL methods are both computationally and memory intensive. This is also very promising for GS since frequently we have more independent variables (p) than sample sizes (n). For example, the ResNet‐50 (residual neural network; He et al., 2015) with 50 convolutional layers needs over 95 MB of memory for storage and over 3.8 billion floating number multiplications when processing an image. After discarding some redundant weights, the network still works as usual but saves more than 75% of parameters and 50% of computational time. However, compression methods are challenging because a good compression method is expected to achieve almost the same prediction performance as the original input data, albeit with much smaller learnable parameters and less computational resources (Cheng et al., 2017).
9 For increasing our efforts for data sharing in platforms to create large data sets for each species containing not only phenotypic and markers data but also environmental information and other omics data. These data sets will be the key to exploit the power of DL methods and to pretrain models that can be used with small data sets.

We suggest addressing all these areas of opportunity for GS research given that there is a practical difficulty in building end‐to‐end DL projects. Because of the inherent complex tuning process, they entail large data sets and considerable computational resources resembling research and development (R&D) more than software development. Usually, this process involves people from multidisciplinary backgrounds, and, therefore, expectations must be grounded. For these reasons, more people need to be involved in these areas of opportunity in addition to allocating more financial resources.

### 2.3 The downside of DL models

Deep‐learning models do not have strong theoretical support because this field is guided by empirical findings rather than a strong theory. Moreover, the loss function only guarantees a local minimum since it is not a convex loss function. However, some authors say that this is not a big issue since almost all local minimums have very similar function value to the global optimum, and hence, finding a local minimum is also good because in many instances recovering the global minimum becomes hard as the network size increases and that in practice is insignificant as global minimum often leads to overfitting (Choromanska et al., 2015). Some of their most fervent adversaries say that DL methods are still alchemy. However, we cannot ignore DL methods’ many successful applications (autonomous cars, home assistants, superhuman Atari game play, AlphaGo, etc.) that are rapidly shaping and changing our world.

Another downside is that the expectations about what DL models are able to achieve are higher than what they really are; empirical evidence suggests that DL models are especially good (better than humans) in narrow tasks with specific instructions trained with large and labeled data sets but are still far from the main goals of artificial intelligence, which Kaplan and Haenlein (2019) define as “a system's ability to correctly interpret external data, to learn from such data, and to use those learnings to achieve specific goals and tasks through flexible adaptation.” This is due to the fact that 99.9% of the applications of DL methods can still be categorized as weak artificial intelligence systems that are far from reaching general intelligence (similar to whole human intelligence) and even farther still from creating superintelligence systems (those that surpass humans in all aspects).

Another unfavorable aspect of DL models is that they come with slogans and straplines used for marketing just as those used for regular commercial products. For this reason, DL methods are overhyped at the moment and the expectations exceed what can be accomplished with this technology. Another downside of DL methods in that they are not easily interpretable, and understanding the decision making in highly sensitive areas such as healthcare, criminology, finance, etc., is of paramount importance. For this reason, a lot of research is needed to be able to help the explainability of DL and many other machine‐learning methods (Burkart & Huber, 2021). There are by now numerous examples of data leakage in DL leading to amazingly performing models that focus on the wrong thing. For example, Wu and Zhang (2016)’s paper purporting to say that they can identify criminals from photos, while it's more likely that the DL algorithm has learned to distinguish smiles from no smiles. Also, there are many devices built using DL that discriminate against people (by age, disability, national origin, race or color, religion, sex, opportunities of employment, etc.) because, in part, training sets are biased.

## 3 DISCUSSION

High‐throughput sequencing technology has brought biological science into a ‘big data’ era with an incomparable explosion of genomic and omics data. For this reason, GS is also entering a new era of petabyte‐level sequencing data. Converting such big data to biological insights presents a huge challenge for computational analysis. Because the use of many hidden layers and the way the neurons communicate (architecture), DL methods are closer to imitating the complexity of biological systems. For this reason, Emmert‐Streib et al. (2020b) point out that these methods are more appropriate for answering complex questions than most statistical machine‐learning methods that can only answer simple questions. Therefore, some of these supervised learning methods are being used in genomics to predict gene expression levels and population structure (Krogel & Scheffer, 2004 ), among others. However, the most sophisticated applications of DL models in biology and other fields are capable of attacking complex questions if they are dissected into smaller problems rather than addressing them as a whole.

As pointed out in some parts of this paper, DL models became popular quite rapidly because of (a) their ability to perform better when solving a number of problems, (b) easier problem solving because of the automated way in which they perform feature engineering, c) they represent all layers simultaneously rather than in many steps, (d) their ability to capture complex nonlinear patterns in the data more efficiently because of the inclusion of many hidden layers, and (e) media hype, enhancing some remarkable achievements.

As was also pointed out, DL methods are not a panacea and should not be blindly adopted. This is especially true if the data set at hand is small and does not have complex nonlinear patterns; under these circumstances, conventional statistical machine‐learning methods used in GS are the best option. For this reason, in this paper we are only encouraging the adoption of DL methods for specific applications in the context of large data sets and complex nonlinear patterns. An advantage is that they do not require extensive feature engineering (preprocessing) of the inputs and are very promising for including many different types of inputs as predictors, which can considerably increase the prediction performance.

The nine areas of opportunities mentioned in this paper for research in DL applied to GS can be justified in part because of the many successful applications of DL in other fields. In addition, most of these published methods have little flexibility when being adapted to new data, like those abundant in GS, and their adaptation requires considerable knowledge and effort. In these nine areas of opportunity, we stress the need for software frameworks that allow for a fast turnover when it comes to addressing new hypotheses, integrating new data sets, or experimenting with new neural‐network architectures. Regarding the architecture, mostly multi‐layer perceptron (fully connected networks) and convolutional neural networks have been used in GS; however, as pointed out in some of the nine areas of opportunity, other DL methods can be explored in GS, such as the use of reinforcement learning, VAE, GAN, and transfer learning, among others. For example, GANs can save on the sequencing cost of a large number of samples.

Although DL methods are not yet the prevalent technique in GS or genomics in general, it has automatic feature extraction ability and greater data representation capability for dealing with high‐dimensional data sets. For these reasons, they are leading innovation and research in fields such as sequence analysis, function prediction, expression prediction, interaction identification, and plant and animal breeding. However, these trends can be empowered in plant breeding by focusing research on the nine areas of opportunity that DL models offer for GS in such a way that we can take full advantage of DL methods.

It must be emphasized that DL is only one of several machine‐learning methods, that is, it is complementary and will not replace conventional statistical machine‐learning methods that are very efficient for small data sets with simple patterns that require little computational resources because the latter are easier to train, understand, and implement. This argument is also supported by the no‐free‐lunch theorem mentioned above that, in summary, “state[s] that any two optimization algorithms are equivalent when their performance is averaged across all possible problems” (Wolpert & Macready, 1997; Wolpert & Macready, 2005). While DL methods are extremely difficult to train, they imitate the structure in visual neuroscience and are able to translate the data representation in an increasingly abstract form by means of nonlinear chunks. Deep‐learning methods have also turned out to be exceptionally successful in learning nonlinear input–output mapping with both increased sagacity and invariance of the representation. That is, DL methods are extremely flexible in the relation assumed between the genomic (markers) information and phenotypic data of traits and are able to efficiently capture complex interaction between genes. Additionally, DL methods perform automatic feature extracting with high selectivity that increases the power of these methods for the analysis of large data sets.

## 4 CONCLUSIONS

Deep‐learning applications are shaping our world because they are helping to increase scientific discovery and the development of technological products to solve complex tasks that can accelerate human progress. However, we perceive that not all researchers working in GS understand what can really be done with DL or have enough clarity on how to build successful data science teams that bring real value to breeding programs using these tools. For this reason, we highlight the power of DL and point out many areas of opportunity that can help its successful adoption for GS. We can see that in this context, because of the availability of large amounts of omics data, the application of DL methods can help to increase the power of GS as well as answer complex questions. Also, there are still DL method tools not used for GS that can help to increase its efficiency; however, to use these tools we must be open to explore these new methods and invest in the required resources to be able to take advantage of this emerging technology that is reshaping and influencing not only our everyday lives but also many other areas of science. For these reasons, we believe breeders should take advantage of these new tools. While we do not expect GS tools to replace breeders, breeders who are more capable of utilizing GS tools will replace those who cannot.

## AUTHOR CONTRIBUTIONS

Osval Antonio Montesinos‐López: Conceptualization; Formal analysis; Writing software, Carlos Moises Hernadez‐Suarez: Conceptualization; Abelardo Montesinos‐López: Conceptualization, Formal analysis; José Alberto Barrón‐López: Conceptualization; Formal analysis; Software, and José Crossa: Conceptualization; Resources; Supervision

## FUNDING

We are thankful for the financial support provided by the Bill & Melinda Gates Foundation [INV‐003439, BMGF/FCDO, Accelerating Genetic Gains in Maize and Wheat for Improved Livelihoods (AG2MW)], the USAID projects [USAID Amend. No. 9 MTO 069033, USAID‐CIMMYT Wheat/AGGMW, AGG‐Maize Supplementary Project, AGG (Stress Tolerant Maize for Africa], and the CIMMYT CRP (maize and wheat). We acknowledge the financial support provided by the Foundation for Research Levy on Agricultural Products (FFL) and the Agricultural Agreement Research Fund (JA) in Norway through NFR grant 267806.

## CONFLICT OF INTEREST

The authors declare no conflicts of interest.
